# YM155 Induces EGFR Suppression in Pancreatic Cancer Cells

**DOI:** 10.1371/journal.pone.0038625

**Published:** 2012-06-18

**Authors:** Young-Soon Na, Soo-Jin Yang, Seung-Mi Kim, Kyung-Ah Jung, Jai-Hee Moon, Jae-Sik Shin, Dok Hyun Yoon, Yong Sang Hong, Min-Hee Ryu, Jae-Lyun Lee, Jung Shin Lee, Tae Won Kim

**Affiliations:** 1 Institute for Innovate Cancer Research, Asan Medical Center, Seoul, Korea; 2 Department of Oncology, Asan Medical Center, University of Ulsan College of Medicine, Seoul, Korea; Technische Universität München, Germany

## Abstract

YM155, which inhibits the anti-apoptotic protein survivin, is known to exert anti-tumor effects in various cancers, including prostate and lung cancer. However, there are few reports describing the inhibitory effect of YM155 on human pancreatic cancers that highly express survivin. Here, we tested the effects of YM155 on a variety of cancer cell lines, including pancreatic cancer cells. We found that YM155 exerts an anti-proliferative effect in pancreatic cancer cells, inducing cell death through suppression of XIAP (X-linked inhibitor of apoptosis) as well as survivin without affecting the anti-apoptotic proteins Bcl-xL or Mcl-1. YM155 also inhibited tumor growth *in vivo*, reducing the size of pancreatic cancer cell line MIAPaCa-2 xenografts by 77.1% on day 31. Western blot analyses further showed that YM155 downregulated phosphoinoside 3-kinase (PI3K) expression and reduced the levels of phosphorylated (activated) extracellular signal-regulated kinase (ERK) and STAT3 (signal transducer and activator of transcription 3) in PANC-1 cells. Interestingly, we also found that YM155 downregulated the epidermal growth factor receptor (EGFR) in various cancer cell lines and induced the EGFR phosphorylation and ubiquitination of EGFR in PANC-1 cells. YM155 also modestly promoted the ubiquitination of survivin and XIAP. Therefore, YM155 acts through modulation of EGFR and survivin expression to subsequently reduce survival. We suggest that YM155 has potential as a therapeutic agent in the treatment of pancreatic cancer.

## Introduction

Pancreatic cancer is a highly aggressive malignant disease. Despite therapeutic advances, the prognosis of patients with pancreatic cancer is extremely poor. The poor prognosis of pancreatic cancer patients is attributable to the tendency of this cancer to respond poorly to chemotherapy. Although the nucleoside analog gemcitabine is currently the standard chemotherapeutic agent used in pancreatic cancer, its survival benefit is unsatisfactory and novel therapeutic agents are desperately needed [1].

The overexpression of survivin, the smallest member (16.5 kDa) of the inhibitor of apoptosis (IAP) family of proteins, has been reported to be important in the development and progression of pancreatic cancer [2], [3]. Survivin contains a single baculovirus IAP repeat (BIR) and interacts with chromosomal passenger proteins, NES (nestin), aurora kinase B, SMAC/DIABLO, CDK1 (cyclin-dependent kinase 1), PKA (protein kinase A), XIAP, and heat shock protein (HSP)-90 [2], [3]. Survivin localizes to the cytoplasm, mitochondria and nuclei, and is expressed ubiquitously in embryonic stages but is undetectable in normal terminally-differentiated adult tissues [2], [3]. Pathologically, survivin is overexpressed in many human cancers, including pancreatic cancer [2], [3]. As survivin is highly expressed in pancreatic tumor tissues, but not in normal pancreatic tissues, it is an attractive target for drug development against pancreatic cancer [4].

YM155, a small molecule inhibitor of survivin developed by Astellas Pharma, Inc. that is currently undergoing clinical trials, suppresses transactivation of survivin through direct binding to its promoter [5]. YM155 has been examined in various cancer cell types, including melanomas and lymphomas, as well as prostate, lung, and breast cancers [6]. Xenograft models have also shown the anticancer efficacy of YM155 in combination with platinum compounds [7] or docetaxel [8] as well as in monotherapy [9].

The epidermal growth factor receptor (EGFR), which is overexpressed in various cancers and has been investigated in relation to cancer prognosis [10], [11], [12], is known to be an important factor in pancreatic cancer progression [13]. The binding of a ligand to EGFR promotes conformational changes that induce receptor dimerization, resulting in tyrosine autophosphorylation and receptor activation. The activated EGFR signals through phosphorylated tyrosine residues to stimulate multiple pathways, including mitogen-activated protein kinase (MAPK), phosphatidylinositol 3-kinase (PI3K)/Akt, signal transducers and activators of transcription (STAT), phospholipase C, and Src/focal adhesion kinase (Src/FAK) pathways. Activation of EGFR signaling plays an important role in fostering proliferation and survival; conversely, interruption of EGFR promotes apoptosis. EGFR-targeted therapeutics, such as erlotinib, have been developed for the treatment of pancreatic cancer.

YM155 has shown antitumor activity in pancreatic cancer cell lines [14]. Moreover, in breast cancer cells, survivin expression has been reported to be regulated by EGFR via the PI3K/AKT pathway, another important pathway in pancreatic cancers [15]. Therefore, it seems possible to have the effect of YM155 on EGFR and its downstream signaling in pancreatic cancer cells because survivin is regulated with EGFR.

In this study, we evaluated the anticancer effects of YM155 in pancreatic cancer cell lines and xenografts. In addition, we investigated the effect of YM155 on EGFR, PI3K, ERK, and STAT3 expression to determine whether YM155 affects the EGFR/PI3K/STAT3 pathways. We also investigated the effect of YM155 on the expression of other members of the IAP family related with anti-apoptotic and pro-apoptotic proteins in pancreatic cancer cell lines.

## Results

### Pancreatic cancer cells are sensitive to YM155

To investigate the anti-proliferative capacity of YM155 in various cancer cell lines, we measured IC_50_ values for YM155 in pancreatic, gastric, and colorectal cancer cells (Table 1 and Table S1). The IC_50_ values for YM155 in pancreatic, gastric, and colorectal cancer cell lines were 3.7–30.3, 1.4–140.8, and 8.7–370.0 nM, respectively. Thus, YM155 is effective in pancreatic, gastric, and colorectal cancer cells.

**Table 1:** YM155 IC_50_ values in pancreatic cancer cell line.

Cell line	YM155 (IC_50_, nM)
PANC-1	3.69
MIAPaCa-2	29.36
BxPC-3	30.26

Pancreatic cancer cell lines were treated with 10^−5^–10^1^ µM YM155 for 48 hours.

**Figure 1:**
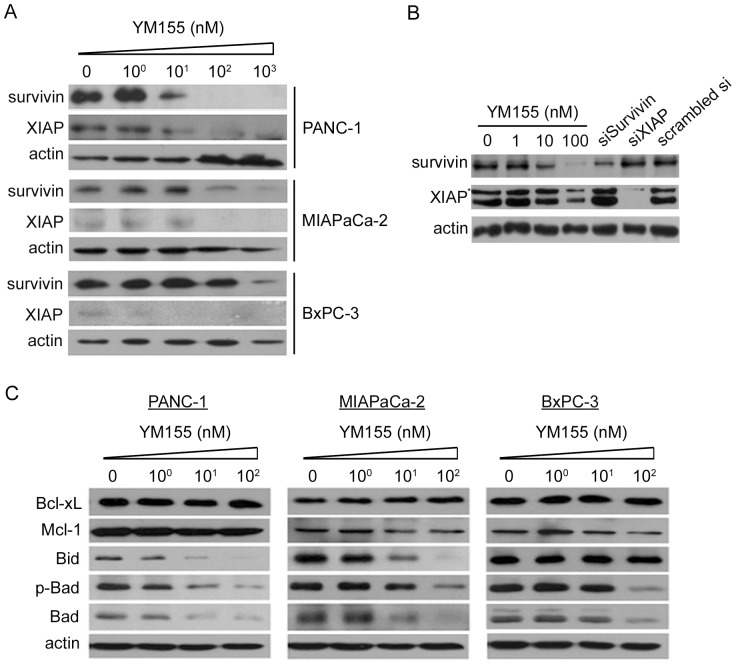
YM155 affects the expression of XIAP as well as survivin in pancreatic cancer cell lines, and inhibits tumor growth in MIAPaCa-2 xenograft tumor models. A, Concentration-dependent effects of a 24-hour treatment with YM155 on the expression of survivin and XIAP in PANC-1, MIAPaCa-2, and BxPC-3 cells were determined by Western blotting. B, The effect of YM155 on the expression of XIAP was compared with that of survivin knockdown by Western blotting. PANC-1 cells were treated with YM155 for 24 hours or transfected with 40 nM siRNA (siSurvivin, siXIAP, and scrambled siRNA) for 48 hours. *, nonspecific. C, Concentration-dependent effects of YM155 on the expression of anti-apoptotic (Bcl-xL and Mcl-1) and pro-apoptotic (Bid and Bad) proteins were examined in PANC-1, MIAPaCa-2, and BxPC-3 cells.

To determine the effect of the survivin inhibitor YM155 on the expression of survivin and XIAP, another member of the IAP family, we performed Western blotting in pancreatic and gastric cancer cell lines (Fig. 1A and Fig. S1). At high concentrations, YM155 significantly decreased the expression of survivin in PANC-1, MIAPaCa-2, BxPC-3, KATOIII, and MKN45 cell lines. YM155 was notably more potent in PANC-1 cells, where it decreased expression of survivin at a low concentration (10 nM). YM155 also inhibited the expression of XIAP in pancreatic and gastric cancer cell lines, except for MKN45 cells; in these cells, YM155 had no discernable effect on XIAP levels. In line with previous observations [9], [16], YM155 showed little effect on the expression of the IAPs, cIAP1 and cIAP2, in pancreatic cancer cell lines (Fig. S2).

To determine whether YM155-induced downregulation of XIAP expression is due to reduced expression of survivin, we knocked down survivin in PANC-1 with siRNA (siSurvivin) and assessed XIAP expression by Western blotting (Fig. 1B). The level of XIAP protein was decreased to a greater extent after exposure to 10 nM YM155 than in cells transfected with siSurvivin, suggesting that the YM155-induced decrease in XIAP levels does not occur via downregulation of survivin. Survivin has been reported to have binding sites for XIAP [2]. To examine the effect of YM155 on survivin–XIAP interactions, we immunoprecipitated survivin and then probed immunoprecipitates by Western blotting with an antibody against XIAP (Fig. S3). Treatment of PANC-1 cells with 100 nM YM155 for 6 hours had no effect on the levels of survivin or XIAP, but did slightly inhibit the interaction of survivin with XIAP, suggesting that YM155 affects the assembly of the survivin–XIAP complex to some extent.

We also investigated the effects of YM155 on the expression of anti-apoptotic and pro-apoptotic proteins in pancreatic cancer cell lines (Fig. 1C). There was no change in Bcl-xL or Mcl-1 levels after treatment with YM155. YM155 induced a concentration-dependent decrease in Bid, p-Bad, and Bad levels in most pancreatic cancer cell lines; the exception was Bid, which was unaffected in BxPC-3 cells.

### YM155 induces downregulation of PI3K, p-ERK, and p-STAT3

**Figure 2:**
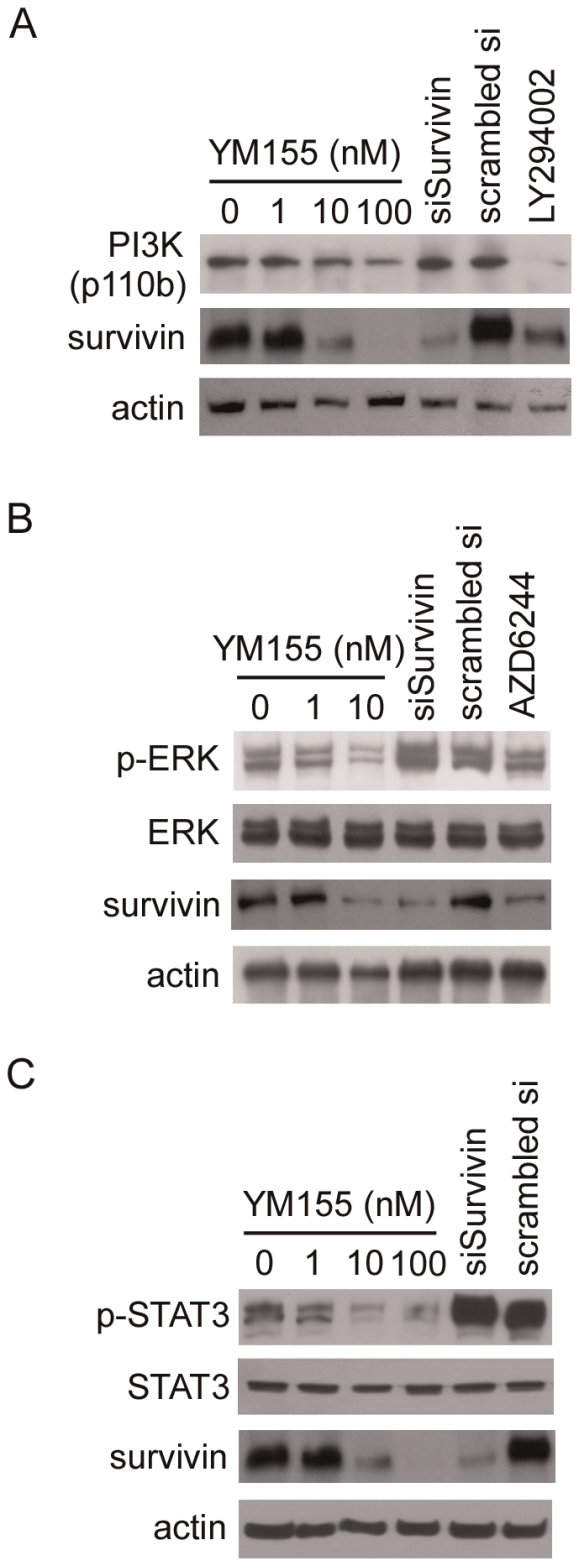
YM155 induces downregulation of PI3K, p-ERK, and p-STAT3 in PANC-1 cells. A, Levels of PI3K and survivin were analyzed by Western blotting after treating with YM155 or 30 µM LY294002 (control) for 24 hours, and 48 hours after transfection with 40 nM siRNA. B, Levels of phosphorylated ERK and expression of survivin were determined by Western blotting after treating for 24 hours with YM155 or 500 ng of AZD6244, and 48 hours after transfection of 40 nM siRNA. C, Levels of phosphorylated STAT3 and expression of survivin were analyzed by Western blotting after a 24-hours treatment with YM155 and 48 hours after transfection of 40 nM siRNA.

Because YM155 suppressed XIAP expression, we examined whether YM155 affected the expression of other signaling components in pancreatic cancer cells. Treatment of HER2-overexpressing MCF7 cells with the PI3K inhibitor LY294002 or ERK1/2 inhibitor U0126 has been reported to downregulate survivin [17]. A STAT3 inhibitor has also been reported to suppress survivin expression [18]. On the basis of these reports, we investigated the effects of YM155 on PI3K, ERK, and STAT3 in PANC-1 cells (Fig. 2), which we had found to be the most YM155-sensitive of the pancreatic cell lines tested. Treatment of these cells with YM155 at concentrations of 10 nM and above decreased PI3K (Fig. 2A), p-ERK (Fig. 2B), and p-STAT3 (Fig. 2C) levels compared with those in siSurvivin-treated cells. The PI3K inhibitor LY294002 and MEK1/2 inhibitor AZD6244 were used as controls to analyze the levels of PI3K, phosphorylated ERK and survivin. We also examined the effects of YM155 on the expression of the PI3K family members p110α, p110β, class III, p85, and p-p85 in PANC-1, MIAPaCa-2, and BxPC-3 cells, and found that all were reduced by 100 nM YM155 in all three cell lines (Fig. S4). Moreover, phosphorylation of Akt, which is a downstream effector of PI3K, was also reduced by YM155 (100 nM) in these pancreatic cancer cell lines. Akt is known to promote cell survival through phosphorylation of Bad [19], [20]. Therefore, a reduction in the phosphorylated form of Akt induces a decrease in p-Bad, as shown in Figure 1C, resulting in cell death.

### YM155 downregulates EGFR expression

**Figure 3:**
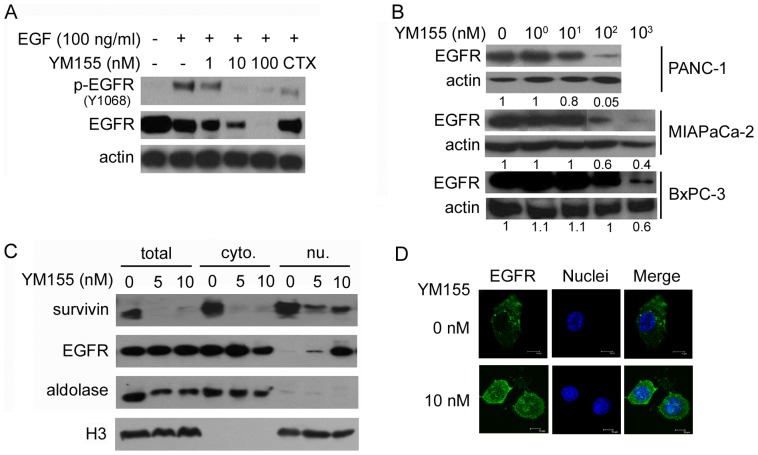
YM155 downregulates EGFR in pancreatic cancer cell lines. A, PANC-1 cells were stimulated with 100 ng/ml EGF for 10 minutes before incubating with or without YM155 for 6 hours. Cetuximab (CTX; 100 ng/ml for 1 hour) was used as a control. Levels of phosphorylated (Y1068) EGFR and total EGFR were analyzed by Western blotting. B, YM155 diminished the expression of EGFR in a concentration-dependent manner in PANC-1, MIAPaCa-2 (cropped blots; full-length blots are presented in Fig. S5A), and BxPC-3 cells. The ratio of EGFR∶actin expression compared with the control is shown for each lane (using Multi-Gauge v2.3 software). C, Survivin and EGFR expression were detected in subcellular fractions of PANC-1 cells by Western blotting. Cytoplasmic (cyto) and nuclear (nu) fractions were isolated after exposure to YM155 for 24 hours. Aldolase (cytoplasmic) and H3 (nuclear) served as markers for the purity of subcellular fractions. Total lysates (total) were analyzed concurrently. D, YM155 (10 nM) induced the nuclear EGFR accumulation. PANC-1 cells were stained with anti-EGFR, anti-Alexa Fluor 488, and DAPI after the YM155 treatment. Scale bars: 10 µm.

Because YM155 downregulates PI3K, p-ERK, and p-STAT3, which are downstream mediators of EGFR signaling [21], [22], [23], we analyzed whether YM155 affects the expression of EGFR (Fig. 3). As expected, treatment with EGF increased EGFR phosphorylation. Interestingly, however, the levels of p-EGFR as well as total EGFR in PANC-1 cells were significantly decreased after exposure to YM155 in the presence of EGF (Fig. 3A). These results suggest that YM155 reduced p-EGFR levels via a decrease in EGFR levels. Cetuximab, used as a positive control, also decreased p-EGFR in the presence of EGF. A further analysis of the effects of YM155 on EGFR expression in various cancer cell lines showed that YM155 induced a concentration-dependent decrease in EGFR levels in pancreatic (PANC-1, MIAPaCa-2, and BxPC-3), colorectal (HCT116), prostate (PC3), and lung (H460) cancer cell lines; similar effects were observed in KATOIII gastric cancer cells but not in MKN45 gastric cancer cells (Fig. 3B and Fig. S5).

We examined the effects of YM155 on survivin and EGFR localization by examining subcellular fraction levels (Fig. 3C); YM155 reduced survivin levels in both cytoplasmic and nuclear fractions of PANC-1 cells. EGFR levels were reduced in the cytoplasmic fraction, but EGFR accumulated in the nuclear fraction. The nuclear EGFR accumulation induced by 10 nM YM155 was confirmed by confocal microscopy (Fig. 3D). Because YM155 (10 nM) had no effect on EGFR levels in total lysates, this condition may reflect nuclear translocation of EGFR. Collectively, these data indicate that YM155 reduces the levels of cytoplasmic and nuclear survivin, and suggest that YM155 promotes EGFR nuclear translocation.

### YM155, which inhibits survivin promoter activity, induces degradation of EGFR, XIAP, and survivin

To determine whether EGFR is the target of YM155 action, we examined the effect of YM155 on PANC-1 cell viability after siRNA-mediated knockdown of EGFR (Fig. S6A). Western blotting revealed that siSurvivin and siEGFR effectively knocked down survivin and EGFR, respectively, as shown by Western blotting, and the percentages of viable cells were approximately 66% and 75%, respectively. The addition of YM155 reduced cell viability in survivin- or EGFR-knockdown cells to a degree similar to that observed in control cells transfected with scrambled siRNA. YM155 acts as a transcriptional inhibitor of survivin, reducing survivin promoter activity in a concentration-dependent manner in PANC-1 cells (Fig. S6B). Because of this inhibition of survivin transcription, YM155 action may be little affected by knockdown of survivin. By extension, the similar reductions in cell viability by YM155 observed in EGFR-knockdown and control (scrambled siRNA) cells may be due to a reduction in survivin level by YM155.

**Figure 4:**
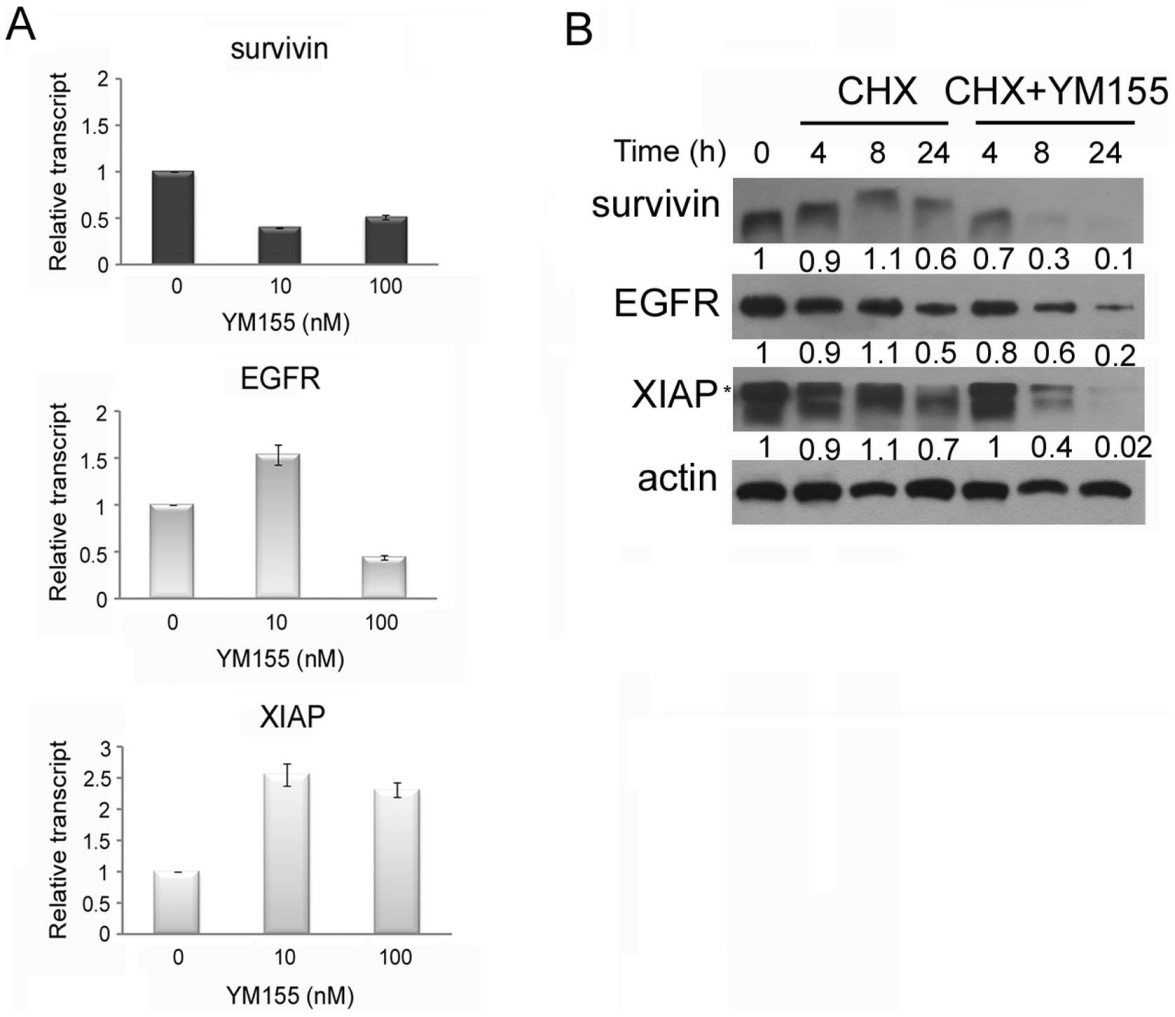
YM155 increases the degradation of survivin, EGFR, and XIAP in PANC-1 cells. A, After treatment of PANC-1 cells with YM155 for 24 hours, survivin, EGFR, and XIAP mRNA levels were determined using real-time RT-PCR. B, Survivin, EGFR, and XIAP levels in PANC-1 cells after treatment with 50 µg/ml cycloheximide (CHX) in the absence or presence of 100 nM YM155 for the indicated periods were examined by Western blotting. The ratio of survivin, EGFR, or XIAP∶actin expression compared with the control is shown for each lane. *, nonspecific.

To determine whether the decreased level of EGFR after YM155 treatment was due to decreased production or increased degradation, we analyzed transcript levels using real-time RT-PCR (Fig. 4A) and assessed the effect of YM155 on protein degradation using the protein synthesis inhibitor cyclohexamide (Fig. 4B) in PANC-1 cells. As expected based on its inhibitory effect on the survivin promoter, YM155 decreased survivin transcript levels. However, XIAP transcript levels were increased, an effect that may be a compensatory response to the decrease in XIAP protein levels. Namely, to protect themselves from apoptosis, cells might upregulate XIAP, another IAP protein, when survivin expression is decreased, but the degradation of XIAP protein by YM155 might result in increased XIAP transcription as a compensatory mechanism. EGFR transcript levels were increased by 10 nM YM155 but were decreased by 100 nM YM155. An analysis of changes in protein levels over time in the absence of protein synthesis (i.e., in the presence of cycloheximide) showed that survivin, EGFR, and XIAP were degraded more rapidly in PANC-1 cells treated with YM155 than in control (cyclohexamide-only) cells. The effects of 100 nM YM155 on the expression of survivin, EGFR, and XIAP in the PANC-1 cells over time were investigated (Fig. S7). The 8 h treatment significantly reduced the survivin levels. Therefore, survivin could be a trigger for subsequent apoptosis. Collectively, these analyses show that YM155 decreased the production of survivin and EGFR (high dose, 100 nM) and enhanced the degradation of survivin, XIAP, and EGFR.

### YM155 induces EGFR phosphorylation, and ubiquitination of EGFR

**Figure 5:**
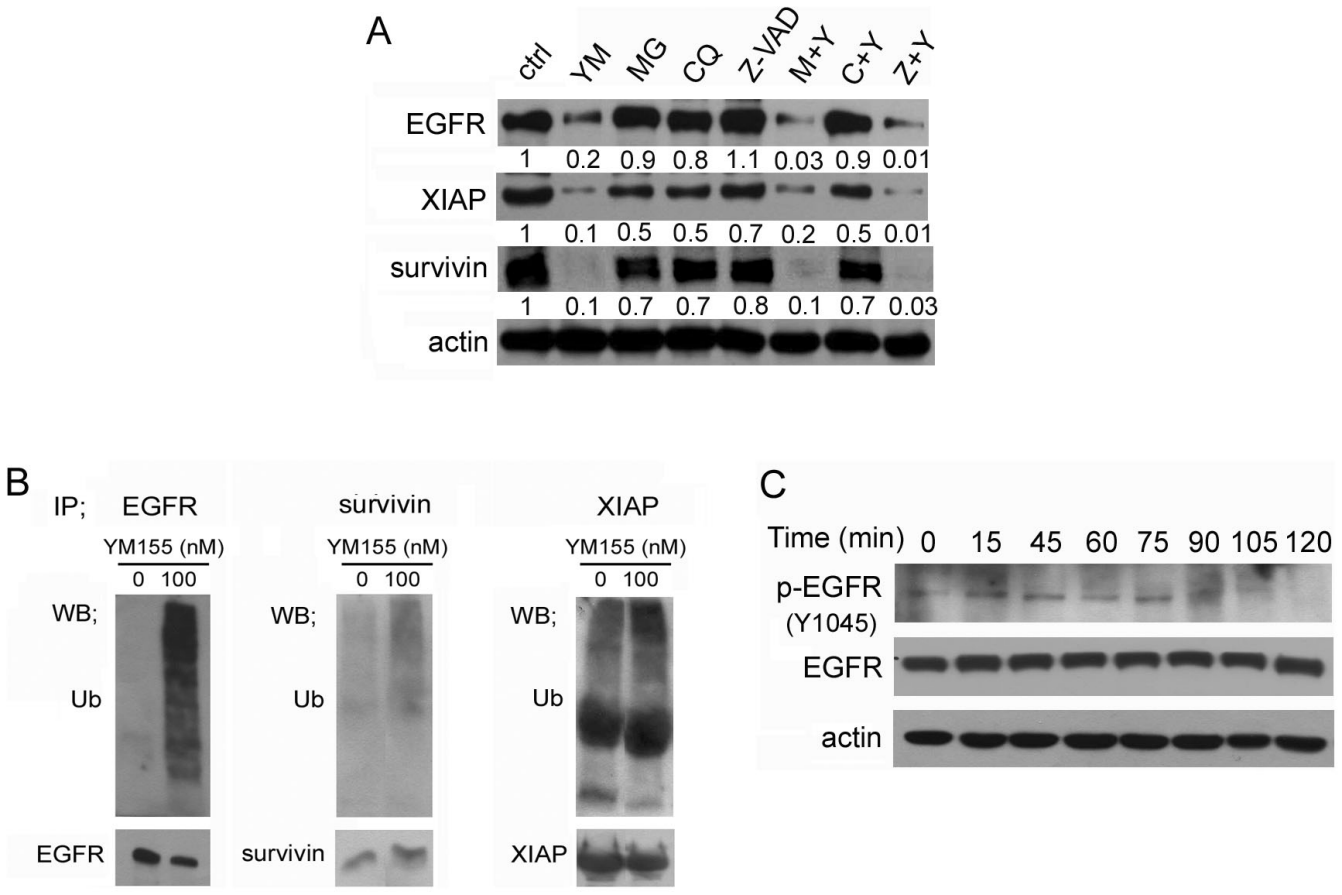
YM155 induces EGFR phosphorylation, EGFR ubiquitination and degradation of EGFR via the lysosome in PANC-1 cells. A, YM155 induced lysosomal degradation of EGFR, XIAP, and survivin in PANC-1 cells. PANC-1 cells were treated with 100 nM YM155 (YM), 10 µM MG-132 (MG), 50 µM chloroquine (CQ), or 30 µM Z-VAD-fmk (Z-VAD) without or with YM155 for 24 hours. The expression ratios of EGFR, XIAP, or survivin to actin compared with the control are shown for each lane. ctrl, control; M+Y, MG-132+YM155; C+Y, chloroquine+YM155; Z+Y, Z-VAD-fmk+YM155. B, PANC-1 cells treated with 100 nM YM155 for 6 hours were immunoprecipitated (IP) with antibodies against EGFR, survivin or XIAP, and analyzed by Western blotting for ubiquitin. C, The time-course of EGFR phosphorylation at Y1045 in PANC-1 cells after treatment with YM155 (100 nM) is shown.

To determine whether YM155 effects on EGFR, XIAP, and survivin degradation were proteasome-, lysosome-, or caspase-dependent, we performed Western blotting in PANC-1 cells after treating with the proteasome inhibitor MG132, the lysosome inhibitor chloroquine, or the caspase inhibitor Z-VAD-fmk (Fig. 5A). Addition of chloroquine to YM155-treated PANC-1 cells blunted the decrease in EGFR, XIAP, and survivin levels, whereas MG132 and Z-VAD-fmk were ineffective, suggesting that the shorter half-life of these proteins in the presence of YM155 was related to enhanced lysosomal degradation. Similar results were observed in MIAPaCa-2 cells (Fig. S8). We next determined whether YM155-induced, lysosome-dependent degradation was associated with ubiquitination of EGFR, XIAP, and survivin (Fig. 5B). Immunoprecipitation experiments revealed a clear increase in ubiquitinated EGFR after treatment with YM155 (100 nM) for 6 hours, and a modest increase in survivin and XIAP ubiquitination. Therefore, YM155 induces ubiquitination of EGFR, survivin, and XIAP.

The Y1045-phosphorylated form of EGFR is known to interact with the E3 ubiquitin-protein ligase c-Cbl, which targets EGFR to the lysosome for subsequent degradation [24]. Because YM155 induced EGFR ubiquitination, we examined the phosphorylation of EGFR as a function of time after treatment with YM155 by probing for pY1045-EGFR by Western blotting (Fig. 5C). These experiments showed a clear decrease in pY1045-EGFR after exposure to YM155 for 105 minutes or more. These results suggest that YM155 modulates Y1045-phosporylation of EGFR.

### YM155 exhibited anti-tumor effects against pancreatic cancer *in vivo*

**Figure 6:**
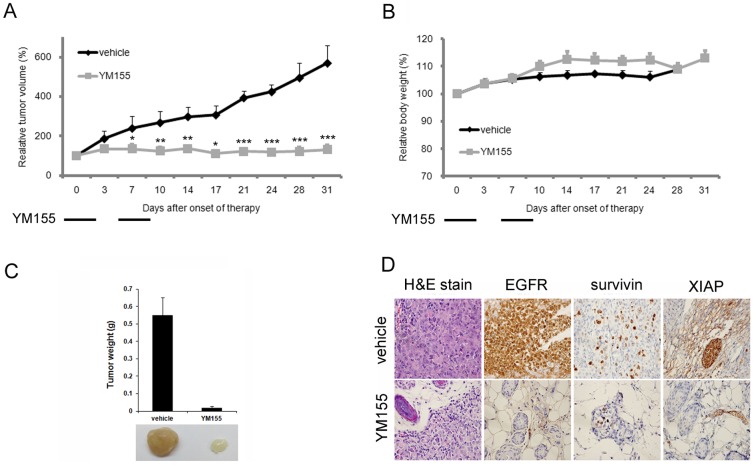
YM155 exhibits anti-tumor effects against pancreatic cancer *in vivo*. Effects of YM155 on tumor growth (A) and body weight (B) *in vivo* were evaluated in MIAPaCa-2 xenografts (vehicle, n = 6; YM155, n = 8). Tumor volume was determined at the indicated times after the onset of treatment. YM155 was administered at dose of 50 mg/kg. The *in vivo* experimental schedule of YM155 is indicated at the bottom of each graph. Day 0 indicates the time at which subcutaneous tumors reached a size of 100 mm^3^ and administration of YM155 was started. Statistical analyses of the effects of YM155 on tumor size were performed using Mann-Whitney tests (*p<0.05, **p<0.01, ***p<0.001, YM155 vs. vehicle). C, Average tumor sizes are depicted for each group on day 31. D, Immunohistochemical analyses in xenograft tumors on day 31 after the YM155 treatment were performed using antibodies against EGFR, survivin, and XIAP and stained with H&E.

To evaluate the anti-tumor effects of YM155 on pancreatic cancer *in vivo*, we employed a MIAPaCa-2 pancreatic cell line xenograft tumor model, measuring tumor volume and body weight (Fig. 6A and 6B). In MIAPaCa-2 xenografts, tumor growth was inhibited by 77.1% on day 31 after treatment with continuous infusion of YM155 (p<0.001, YM155 vs. vehicle). Body weight was not significantly different between untreated and YM155-treated groups. YM155-treated group on day 31 had smaller tumor weights than the vehicle-treated animals (Fig. 6C). YM155 induced the downregulation of EGFR, survivin, and XIAP expressions in xenograft tumors (Fig. 6D). These results indicate that YM155 exerts a significant anti-tumor effect against pancreatic cancer *in vivo*.

## Discussion

In the present study, we investigated the effects of the survivin inhibitor YM155 on survivin, XIAP, and EGFR expression in pancreatic cancer cell lines. Our data show that YM155 affects the expression of both survivin and EGFR and thereby reduces survival. Although previous studies have suggested that YM155 specifically blocks survivin expression in prostate cancer cells and non-small cell lung cancer (NSCLC) cells without affecting other proteins [9], [16], our data from pancreatic and gastric cancer cells except MKN45 cells suggest otherwise. However, like its effects in prostate cancer or NSCLC cells, YM155 effects on XIAP expression in MKN45 cells were almost not detected. Such cell-type differential expression of some proteins might reflect cell-line specific responses of pancreatic or gastric cancer cells to YM155. In line with previous observations, we found that PANC-1 cells were the most susceptible to YM155 among the pancreatic cancer cell lines [6]. PANC-1 cells are known to express higher survivin mRNA levels than the other two pancreatic cancer cell lines [25]. Buck et al. reported that EGFR expression level is higher in PANC-1 cells than in MiaPaCa-2 cells [25]. PANC-1 cells expressing high levels of total and activated AKT have been reported to exhibit significant cell cycle arrest and apoptosis when PI3K/AKT is inhibited, in comparison with the other two pancreatic cancer cell lines [26], [27]. PI3K/AKT is regulated by EGFR [28]. Therefore, the effect of YM155 on EGFR in PANC-1 cells might be associated with AKT. In addition, the high expression of survivin and EGFR might be cytoprotective and correlate with YM155 susceptibility. However, further study is needed to investigate whether susceptibility of cells to YM155 is associated with cell-line specific alterations in genes such as EGFR or survivin. In this study, we have shown that YM155 inhibits tumor growth in MIAPaCa-2 xenografts Therefore, the combination of YM155 with gemcitabine is also worthy of investigation in pancreatic cancer.

YM155 is currently undergoing clinical trials [29]. Phase II trials of YM155 suggested the activity and tolerability in patients with melanoma and advanced refractory non-small cell lung cancer [30], [31]. The YM155 doses tested in the preclinical stages through the determination of the maximum tolerated dose (MTD) or pharmacokinetics studies are applicable to the clinical settings. A clinical trial can be designed on the basis of the *in vitro* and *in vivo* efficacy of YM155. Based on our preclinical data, YM155 is worth investigating further in patients with pancreatic cancer.

In our study, YM155 did not change Bcl-xL or Mcl-1 levels. The results for Bcl-xL were in line with previous observations [9], whereas those for Mcl-1 were in contrast with those of a previous report [32]. Tang et al. reported that YM155 downregulates Mcl-1 in various cancer cell types, but not in pancreatic cancer cells. This might reflect the cell line-specific responses of pancreatic cancer cells to YM155. While YM155 induced a concentration-dependent decrease in Bid, p-Bad, and Bad levels in most pancreatic cancer cell lines, Bid was unaffected in BxPC-3 cells. These results indicate that YM155 affects apoptotic proteins levels, a result that contrasts with previous reports [9].

Previous study showed that a phosphorylation of EGFR was induced by ionizing radiation in a ligand-independent manner and ionizing radiation or cisplatin without EGF induced EGFR transport into the nucleus [33]. They showed that the mechanism for radiation-induced EGFR import into the nucleus was associated with a karyopherin α. However, we do not know the exact mechanism of nuclear translocation of EGFR by YM155 without EGF yet. Thus, further studies are needed to find out the mechanism by YM155 in nuclear translocation of EGFR. Liccardi et al. reported that nuclear translocation of EGFR is important in modulating the repair of DNA damage following chemotherapy [34]. In this study, 10 nM YM155 induced nuclear translocation of EGFR and increased EGFR transcript levels. EGFR translocates to the nucleus, where EGFR might activate genes associated with repair as a transcription factor [35]. However, higher concentrations of YM155 (100 nM) reduced EGFR transcript levels and enhanced EGFR degradation. Therefore, increased transcription and translocation of EGFR at low concentrations (10 nM) of YM155 might protect cells from apoptosis, whereas high concentrations (100 nM) decrease cell survival by reducing EGFR transcription and increasing EGFR degradation.

Levkowitz et al. reported that binding of EGF to EGFR causes EGFR degradation through binding with c-Cbl at the pY1045-EGFR [36]. Ahsan et al. reported EGFR phosphorylation, ubiquitination and degradation in cisplatin-induced cytotoxicity [37]. Pangbum et al reported that sulindac metabolite also induces the ubiquitination of EGFR [38]. Similarly, we found that EGFR phosphorylation, and EGFR ubiquitination and degradation after treatment with YM155 were induced. However, additional research is needed to investigate E3 ubiquitin ligase to YM155.

XIAP has been reported to induce the downregulation of survivin through XAF1 (XIAP associated factor 1) [39]. XIAP has also been identified as a cofactor of survivin in the inhibition of apoptosis [40]. Survivin released from mitochondria in response to apoptotic stimuli interacts with XIAP through an XIAP-binding site corresponding to Lys15-Met38, resulting in increased XIAP stability against ubiquitination/proteasomal degradation and inhibition of apoptosis [41], [42]. Phosphorylation of survivin in the cytoplasm inhibits the assembly of the survivin–XIAP complex, abolishing its anti-apoptotic function [41]. Our results showed that the effect of YM155 on XIAP expression differed in the context of survivin knockdown. YM155 induced an increase in XIAP transcript levels and promoted XIAP protein degradation. YM155 decreased the interaction of survivin with XIAP, slightly enhanced ubiquitination of XIAP, and induced lysosomal degradation of XIAP. Therefore, YM155 affects the degradation of XIAP as well as survivin, and interferes with the assembly of the survivin–XIAP complex. The YM155-induced decrease in XIAP levels is unlikely due to a reduction in survivin levels. In this study, we did not examine phosphorylation of survivin by YM155 or investigate other factors that might affect the survivin–XIAP complex. Accordingly, additional in-depth mechanistic studies on YM155 modulation of XIAP should be performed.

In conclusion, we found that YM155, known as a survivin inhibitor, promotes downregulation of PI3K, p-ERK, and p-STAT3 through degradation of EGFR in pancreatic cancer cells. Our data suggest that YM155 has therapeutic potential in pancreatic cancer and provide support for clinical trials of YM155 in this context.

## Materials and Methods

### Cell lines, compounds, plasmid, and antibodies

The human pancreatic cancer cell lines PANC-1, MIAPaCa-2, and BxPC-3 were obtained from the American Type Culture Collection (Manassas, VA, USAA). YM155 was obtained from Hanmi Pharmaceuticals (Seoul, Korea). Other reagents used include LY294002 (Calbiochem, Darmstadt, Germany), AZD6244 (Chemizon Korea), MG132 (Calbiochem), chloroquine (Sigma-Aldrich, St Louis, MO), Z-VAD-fmk (R&D Systems, MN, USA), cycloheximide (Sigma-Aldrich), epidermal growth factor (EGF; Daewoong Pharmaceuticals, Korea), and cetuximab (Merck Korea). Primary antibodies against the following proteins were used for Western blot analyses: survivin (Abcam, Cambridge, MA, USA); XIAP (BD Biosciences, San Jose, CA, USA); ubiquitin and β-actin (Santa Cruz Biotechnology, Santa Cruz, CA); EGFR, phospho (p)-EGFR (Y1068, Y1045), Bcl-xL, Mcl-1, Bid, p-Bad (S112), Bad, PI3K, ERK, p-ERK (T202/Y204), STAT3, p-STAT3 (Y705), aldolase, and H3 (Cell Signaling, Danvers, MA, USA). Immunoreactive proteins were detected using species-appropriate horseradish peroxidase-conjugated secondary antibodies. Some materials are described in Materials and Methods S1.

### Cell viability assay and siRNA transfection

Cell viability was determined using a Cell Counting Kit-8 (Dojindo Laboratories, Kumamoto, Japan), according to the manufacturer's instructions. Three independent experiments were performed in duplicate. Fifty percent inhibitory concentrations (IC_50_s) were determined using GraphPad Prism (Graphpad Software Inc., San Diego). siRNA against survivin (siSurvivin, 5′-AAG GCU GGG AGC CAG AUG ACG UU-3′ [sense]; 5′-CGU CAU CUG GCU CCC AGC CUU UU-3′ [antisense]), XIAP (siXIAP, 5′-AAG UGG UAG UCC UGU UUC AGC U-3′ [sense]; 5′-GCU GAA ACA GGA CUA CCA CUU UU-3′ [antisense]), EGFR (siEGFR, 5′-AAG AUC AUA AUU CCU CUG CUU-3′ [sense]; 5′-GCA GAG GAA UUA UGA UCU UUU-3′ [antisense]), and scrambled siRNA (control, non-targeting siRNA) were obtained from Thermo Scientific Dharmacon (Lafayette, CO, USA). siRNA transfections were carried out using Lipofectamine RNAiMAX (Invitrogen, San Diego, CA, USA), according to the manufacturer's protocols.

### Real-time reverse transcription-polymerase chain reaction (RT-PCR) analysis

Total RNA from PANC-1 cells treated with YM155 (0, 10, 100 nM) for 24 hours was isolated using TRIzol reagent (Invitrogen), and cDNA was synthesized from total RNA using the SuperScript III First-Strand Synthesis System (Invitrogen). Quantitative PCR was then performed on a LightCycler 480 instrument (Roche, Germany) using SYBR Green I Master (Roche) and the following primers (Xenotech, Daejeon, Korea): survivin, 5′-ATT CGT CCG GTT GCG CTT TCC-3′ (forward) and 5′-CAC GGC GCA CTT TCT TCG CAG-3′ (reverse); EGFR, 5′-CTC AGC CAC CCA TAT GTA CC-3′ (forward) and 5′-CGT CCA TGT CTT CTT CAT CC-3′ (reverse); XIAP, 5′-GAC AGT ATG CAA GAT GAG TCA AGT CA-3′ (forward) and 5′-GCA AAG CTT CTC CTC TTG CAG-3′ (reverse); and GAPDH, 5′-GAG TCA ACG GAT TTG GTC GT-3′ (forward) and 5′-TTG ATT TTG GAG GGA TCT CG-3′ (reverse). A melting curve analysis was performed on all PCR products to confirm the specificity of amplification reactions.

### Immunoprecipitation and subcellular fractionation

Following treatment of PANC-1 cells with 100 nM YM155, whole-cell lysates were prepared in lysis buffer (20 mM Tris, 150 mM sodium chloride, 1 mM ethylenediaminetetraacetic acid, 1 mM ethylene glycol tetraacetic acid, 2.5 mM sodium pyrophosphate, 1 mM β-glycerol phosphate, 0.5% Triton X-100) containing protease inhibitor cocktail (Roche Germany) and phosphatase inhibitor cocktail (Sigma-Aldrich). For immunoprecipitations, pre-cleared cell lysates (500 µg protein) were incubated overnight at 4°C with primary antibodies against survivin, EGFR, or XIAP (Abnova, Fig. 5B) precoupled to protein G sepharose (GE Healthcare, Piscataway, NJ). After five washes with washing buffer (10 mM Tris-Cl pH 7.4, 200 mM sodium chloride, 2.5 mM MgCl_2_, 0.1% Triton X-100, protease inhibitor cocktail), immunocomplexes were examined by Western blotting using antibodies indicated in the text. Nuclear and cytoplasmic extracts were prepared using a NE-PER Nuclear and Cytoplasmic Extraction Kit (Pierce, Rockford, IL, USA), according to manufacturer's protocols.

### Immunofluorescence

PANC-1 cells treated for 24 hours with YM155 were fixed with a 4% paraformaldehyde solution and permeabilized with 0.2% Triton X-100 in PBS. And then, cells were blocked with 10% fetal bovine serum (FBS) and incubated at 4°C for 18 hours with anti-EGFR (Calbiochem, San Diego, CA, USA) in 1.5% FBS in PBS. Alexa Fluor 488 anti-mouse (Molecular Probes and Invitrogen) was incubated at room temperature for 1 hour. Finally, cells were stained with DAPI (Invitrogen) for 5 min and mounted with fluorescence mounting medium (Dako, Carpinteria, CA, USA). Cells were visualized by confocal microscopy (Leica, Wetzlar, Germany).

### Xenograft model

Five-week-old female athymic nude (nu/nu) mice were purchased from Japan SLC Inc. (Shizuoka, Japan). The study was approved by our Institutionl Animal Care and Use Committee (IACUC). Tumors were established by injecting 5×10^6^ MIAPaCa-2 cells subcutaneously into the left flank of mice. When subcutaneous tumors reached a size of 100 mm^3^ (day 0), xenografted animals were randomly allocated into vehicle and YM155 (50 mg/kg) groups. YM155 was subcutaneously administered as a 3-day continuous infusion per week for 2 weeks using an implanted microosmotic pump (Alzet model 1003D, Durect) [9]. Tumors were measured twice weekly with calipers, and volume (mm^3^), calculated as (length×width^2^)/2, was determined. Body weights were also monitored.

### Histopathology and immunohistochemistry

Tumor samples were fixed with 10% formalin and embedded in paraffin, and 4 µm sections were prepared. 4 µm sections were stained with hematoxylin and eosin (H&E). The overexpressions of EGFR, survivin, or XIAP were examined immunohistochemically in tissues from mice bearing MIAPaCa-2 xenograft tumors. Immunohistochemical analyses were performed as previously described [15].

### Statistical Analysis

The data obtained are expressed as means ± SEMs. Differences between tests groups were analyzed by Mann-Whitney tests using GraphPad InStat (Graphpad Software). *P*-values <0.05 were considered statistically significant.

## Supporting Information

Figure S1 **YM155 affects expression of XIAP as well as survivin in gastric cancer cell lines.** Concentration-dependent effects of 24-hour treatment with YM155 on the expression of survivin and XIAP in KATOIII and MKN45 cells were determined by Western blotting.
(TIF)

Figure S2 **YM155 has little effect on the expression of cIAP1/2 in PANC-1, MIAPaCa-2, and BxPC-3 cells.** Cells were treated with different concentrations of YM155 for 24 hours.
(TIF)

Figure S3 **Survivin–XIAP interactions are affected by YM155.** Following treatment of PANC-1 cells with 100 nM YM155 for 6 hours, cell lysates were immunoprecipitated with an anti-survivin antibody and immunoprecipitates were probed for XIAP and survivin by Western blotting. The levels of survivin and XIAP in YM155-treated PANC-1 cell lysates are shown. The ratio of XIAP∶survivin expression compared to control (0 nM YM155) is shown for each lane (using Multi-Gauge v2.3 software). NC, negative control (no lysates).
(TIF)

Figure S4 **YM155 decreases the expression of PI3K family members and p-Akt levels in pancreatic cancer cell lines.** Western blotting was performed after treatment of three pancreatic cancer cell lines with different concentrations of YM155 for 24 hours.
(TIF)

Figure S5 **YM155 downregulates expression of EGFR in various cancer cell lines.** A, Full-length blots showing concentration-dependent effects of YM155 on EGFR expression in MIAPaCa-2 are presented. B. YM155 diminished the expression of EGFR in a concentration-dependent manner in HCT116 and KATOIII cells, but not in MKN45 cells. In PC3 prostate cancer cells (C) and H460 lung cancer cells (D), YM155 decreased the expression of EGFR in a concentration-dependent manner, but did not change the expression of XIAP as in previous reports [9], [16].
(TIF)

Figure S6 **YM155 decreases survivin transcriptional activity in PANC-1 cells.** A, The viability of PANC-1 cells was examined after transfection with siSurvivin, siEGFR, or scrambled siRNA (40 nM) for 48 hours followed by incubation without or with YM155 (10 nM) for 24 hours. B, YM155 induces a concentration-dependent decrease in survivin transcriptional activity in PANC-1 cells, measured using a survivin gene promoter-driven luciferase reporter.
(TIF)

Figure S7 **Treatment of YM155 for 8 hours significantly reduces survivin levels.** Effects of 100 nM YM155 on the expression of survivin, EGFR, and XIAP in PANC-1 cells according to time were determined by Western blotting.
(TIF)

Figure S8 **YM155 induces the lysosomal degradation of EGFR, XIAP, and survivin in MIAPaCa-2 cells.** MIAPaCa-2 cells were treated with 100 nM YM155 (YM), 10 µM MG-132 (MG), 50 µM chloroquine (CQ), or 30 µM Z-VAD-fmk (Z-VAD) without or with YM155 for 24 hours. ctrl, control; M+Y, MG-132+YM155; C+Y, chloroquine+YM155; Z+Y, Z-VAD-fmk+YM155.
(TIF)

Materials and Methods S1 **Cell lines, compounds, plasmid, and antibodies. Transfection and luciferase reporter assay.**
(DOC)

Table S1 **YM155 IC_50_ values in gastric and colorectal cancer cell lines.**
(DOC)
