# Supplementary figures and images for: YM155 Induces EGFR Suppression in Pancreatic Cancer Cells
Source: PLoS One. 2012 Jun 18;7(6):e38625. doi: 10.1371/journal.pone.0038625 (PMC3377633; doi:10.1371/journal.pone.0038625)

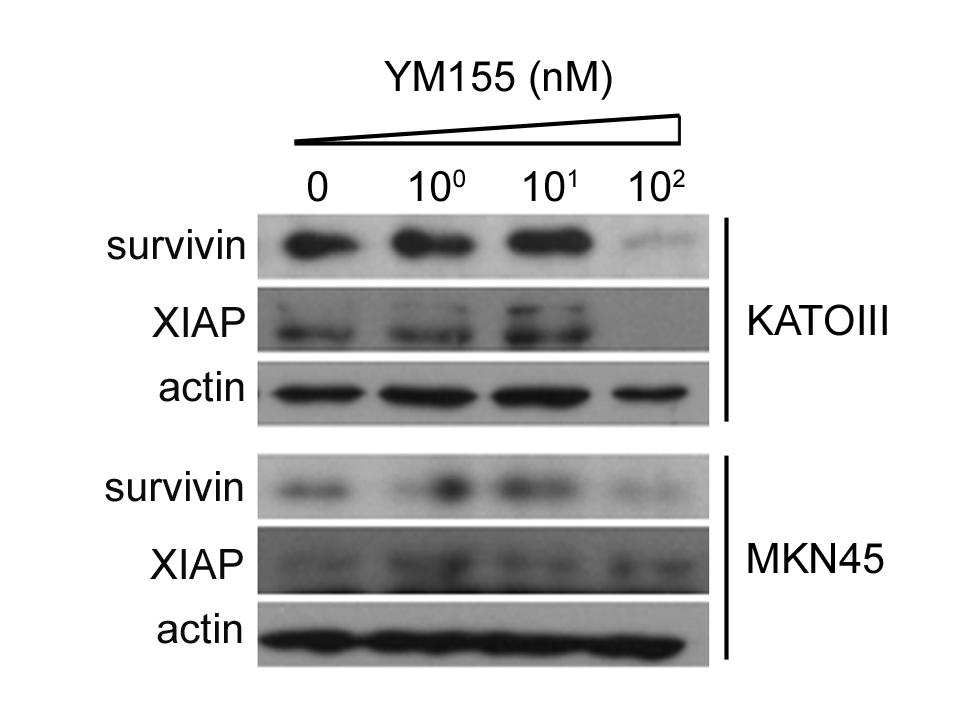

Supplement: Figure S1 — YM155 affects expression of XIAP as well as survivin in gastric cancer cell lines. Concentration-dependent effects of 24-hour treatment with YM155 on the expression of survivin and XIAP in KATOIII and MKN45 cells were determined by Western blotting. (TIF) [file pone.0038625.s001.tif]

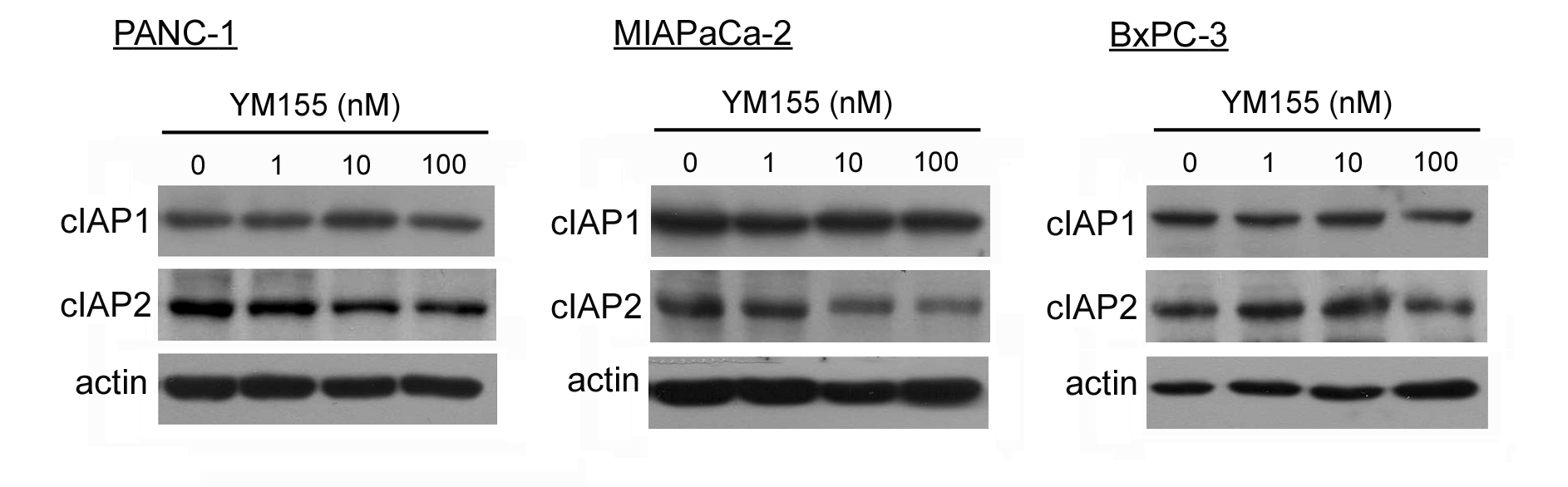

Supplement: Figure S2 — YM155 has little effect on the expression of cIAP1/2 in PANC-1, MIAPaCa-2, and BxPC-3 cells. Cells were treated with different concentrations of YM155 for 24 hours. (TIF) [file pone.0038625.s002.tif]

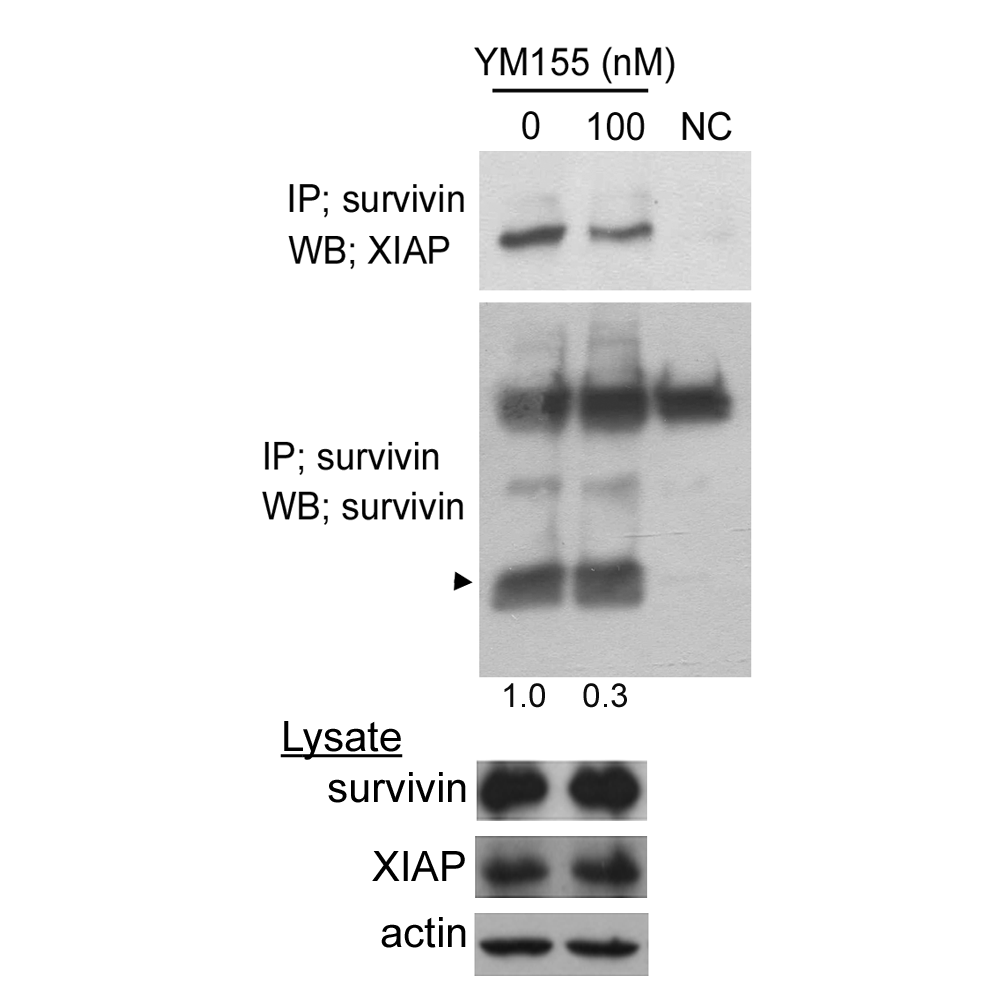

Supplement: Figure S3 — Survivin–XIAP interactions are affected by YM155. Following treatment of PANC-1 cells with 100 nM YM155 for 6 hours, cell lysates were immunoprecipitated with an anti-survivin antibody and immunoprecipitates were probed for XIAP and survivin by Western blotting. The levels of survivin and XIAP in YM155-treated PANC-1 cell lysates are shown. The ratio of XIAP∶survivin expression compared to control (0 nM YM155) is shown for each lane (using Multi-Gauge v2.3 software). NC, negative control (no lysates). (TIF) [file pone.0038625.s003.tif]

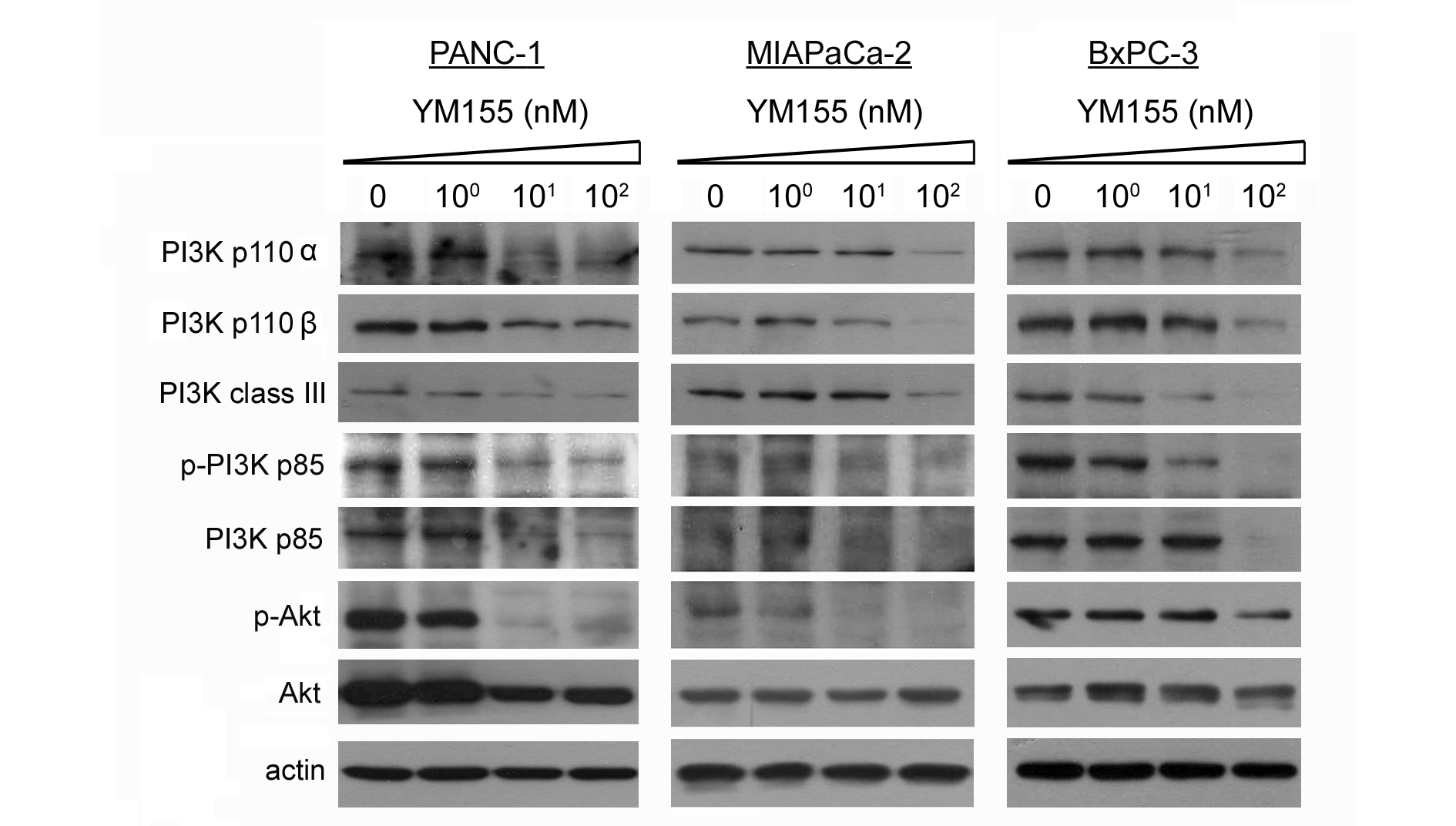

Supplement: Figure S4 — YM155 decreases the expression of PI3K family members and p-Akt levels in pancreatic cancer cell lines. Western blotting was performed after treatment of three pancreatic cancer cell lines with different concentrations of YM155 for 24 hours. (TIF) [file pone.0038625.s004.tif]

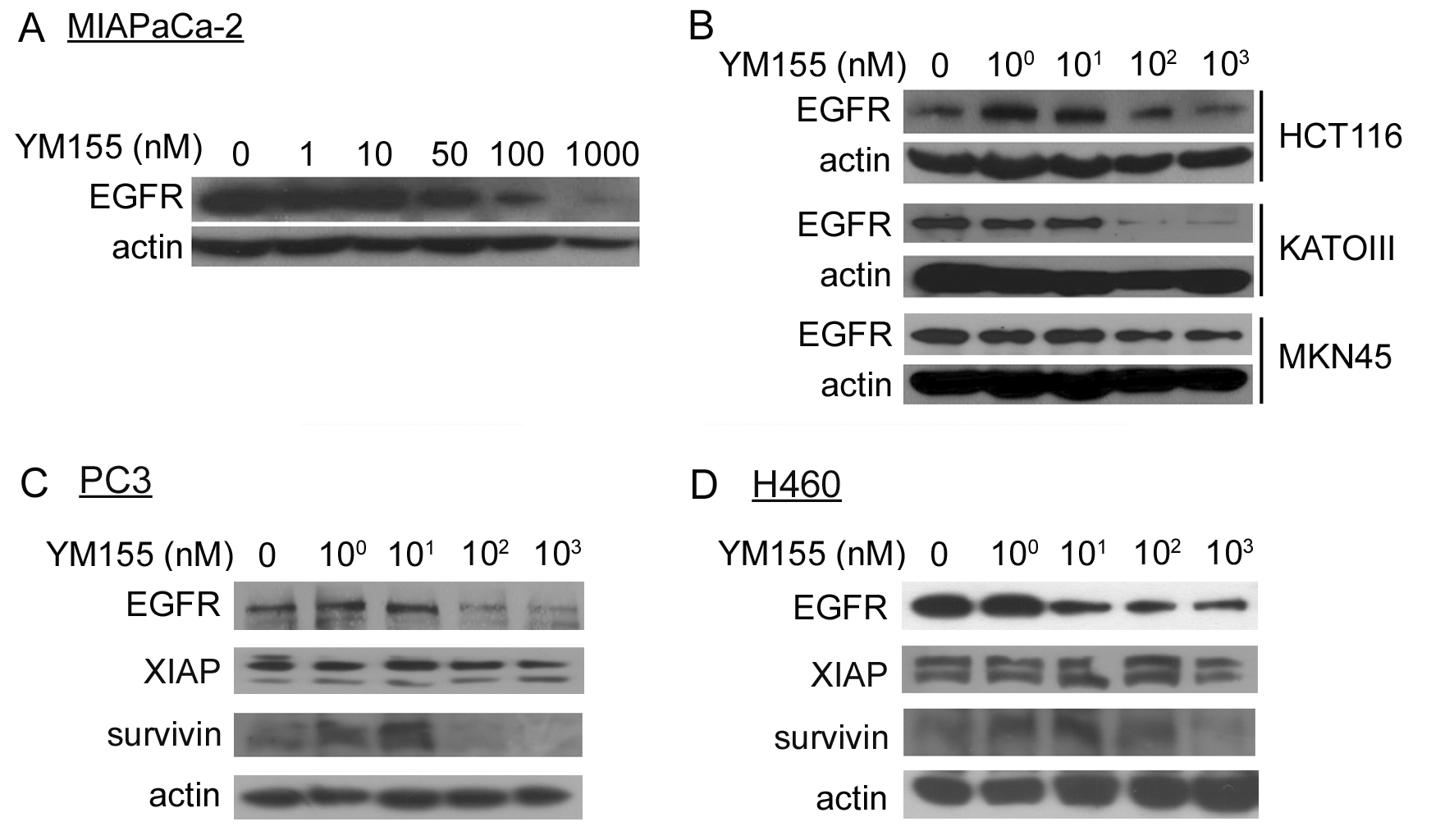

Supplement: Figure S5 — YM155 downregulates expression of EGFR in various cancer cell lines. A, Full-length blots showing concentration-dependent effects of YM155 on EGFR expression in MIAPaCa-2 are presented. B. YM155 diminished the expression of EGFR in a concentration-dependent manner in HCT116 and KATOIII cells, but not in MKN45 cells. In PC3 prostate cancer cells (C) and H460 lung cancer cells (D), YM155 decreased the expression of EGFR in a concentration-dependent manner, but did not change the expression of XIAP as in previous reports [9], [16]. (TIF) [file pone.0038625.s005.tif]

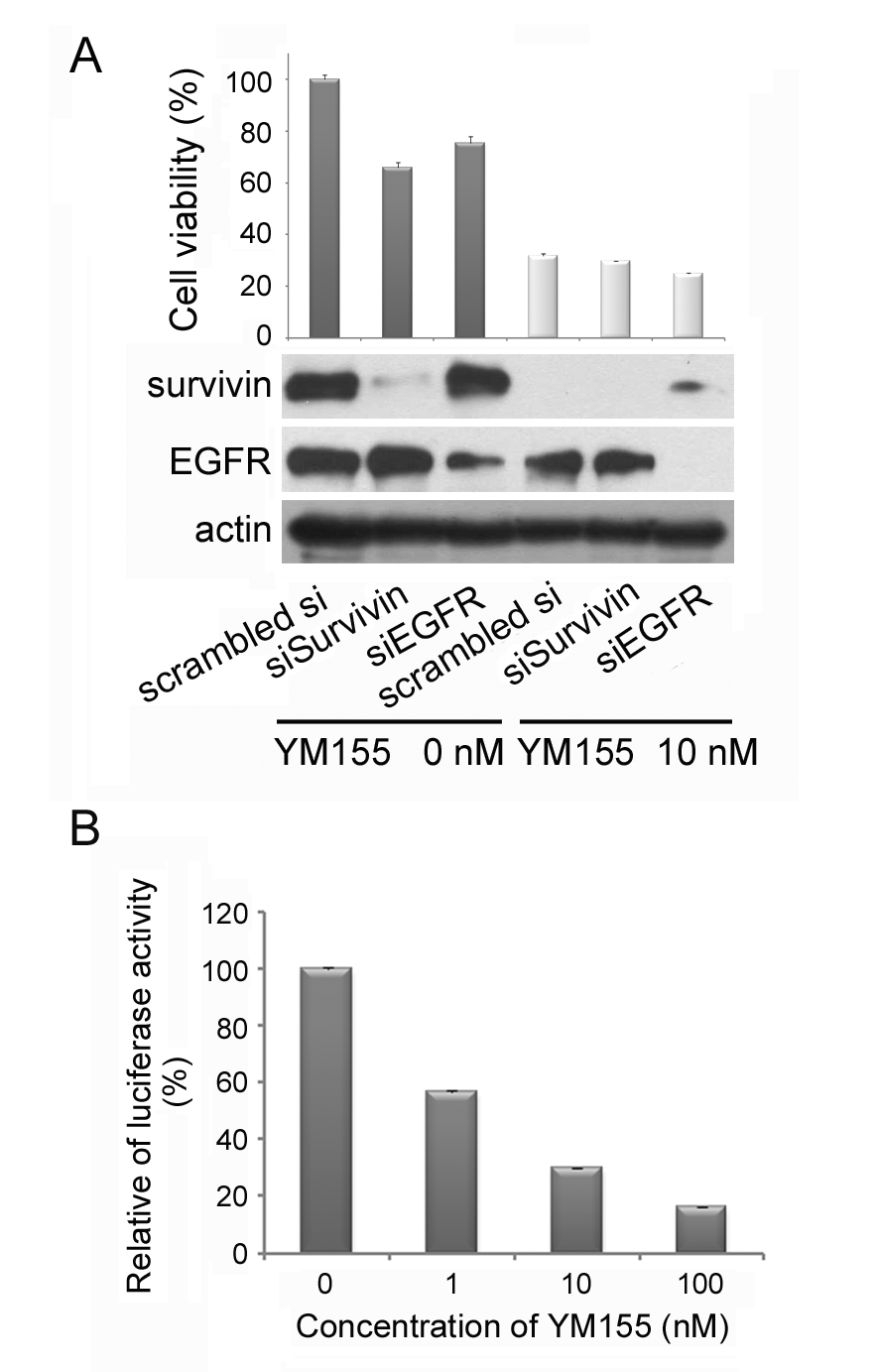

Supplement: Figure S6 — YM155 decreases survivin transcriptional activity in PANC-1 cells. A, The viability of PANC-1 cells was examined after transfection with siSurvivin, siEGFR, or scrambled siRNA (40 nM) for 48 hours followed by incubation without or with YM155 (10 nM) for 24 hours. B, YM155 induces a concentration-dependent decrease in survivin transcriptional activity in PANC-1 cells, measured using a survivin gene promoter-driven luciferase reporter. (TIF) [file pone.0038625.s006.tif]

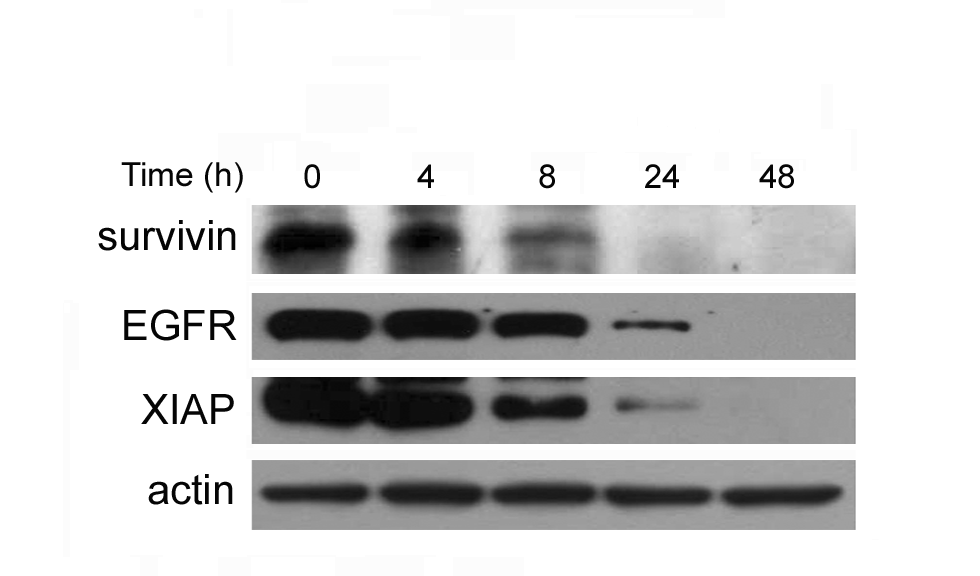

Supplement: Figure S7 — Treatment of YM155 for 8 hours significantly reduces survivin levels. Effects of 100 nM YM155 on the expression of survivin, EGFR, and XIAP in PANC-1 cells according to time were determined by Western blotting. (TIF) [file pone.0038625.s007.tif]

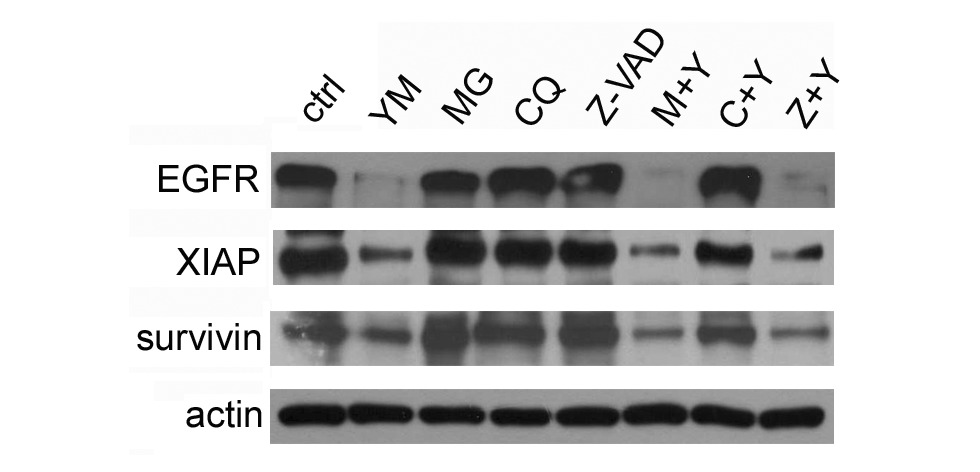

Supplement: Figure S8 — YM155 induces the lysosomal degradation of EGFR, XIAP, and survivin in MIAPaCa-2 cells. MIAPaCa-2 cells were treated with 100 nM YM155 (YM), 10 µM MG-132 (MG), 50 µM chloroquine (CQ), or 30 µM Z-VAD-fmk (Z-VAD) without or with YM155 for 24 hours. ctrl, control; M+Y, MG-132+YM155; C+Y, chloroquine+YM155; Z+Y, Z-VAD-fmk+YM155. (TIF) [file pone.0038625.s008.tif]
