# Supplementary material for: YM155 Induces EGFR Suppression in Pancreatic Cancer Cells
Source: PLoS One. 2012 Jun 18;7(6):e38625. doi: 10.1371/journal.pone.0038625 (PMC3377633; doi:10.1371/journal.pone.0038625)
Supplement: Materials and Methods S1 — Cell lines, compounds, plasmid, and antibodies. Transfection and luciferase reporter assay. (DOC) [file pone.0038625.s009.doc]

**Supporting Information**

**Materials and Methods S1**

**Cell lines, compounds, plasmid, and antibodies**

The human colon cancer cell lines HCT116, HT29, SW620, and HCT15, the human gastric cancer cell lines KATOIII and NCI-N87, the human prostate cancer cell line PC3, and the human lung cancer cell line H460 were obtained from the American Type Culture Collection (Manassas, VA, USA). The human gastric cancer cell line MKN45 was obtained from the Japanese Riken Cell Bank (Tsukba, Japan). The human gastric cancer cell lines SNU484 and SNU620 were obtained from the Korean Cell line Bank (Seoul, Korea). The survivin gene promoter-driven luciferase reporter plasmid pLuc-1458neo was obtained from Hanmi Pharmaceuticals. For Western blot analyses, antibodies against cIAP-1/2, PI3K (p110α, p110β, classIII, p-p85 (Y458), and p85), p-Akt (S473), and Akt were purchased from Cell Signaling (Danvers, MA, USA).

**Transfection and luciferase reporter assay**

pLuc-1458neo transfections were carried out using Lipofectamine LTX and PLUS Reagents (Invitrogen, San Diego, CA, USA), according to the manufacturer’s protocols. Forty-eight hours after transfection with pLuc-1458neo, luciferase activity in PANC-1 cells was assessed using the Luciferase Assay System (Promega, Madison, WI, USA).
