# Supplementary material for: YM155 Induces EGFR Suppression in Pancreatic Cancer Cells
Source: PLoS One. 2012 Jun 18;7(6):e38625. doi: 10.1371/journal.pone.0038625 (PMC3377633; doi:10.1371/journal.pone.0038625)
Supplement: Table S1 — YM155 IC50 values in gastric and colorectal cancer cell lines. (DOC) [file pone.0038625.s010.doc]

**Table S1.** YM155 IC_50_ values in gastric and colorectal cancer cell lines.

| Type | Cell line | YM155 (IC_50_, nM) |
| --- | --- | --- |
| Gastric | KATOIII | 12.48 |
|  | MKN45 | 65.45 |
|  | NCI-N87 | 36.75 |
|  | SNU484 | 1.35 |
|  | SNU620 | 140.80 |
| Colorectal | HCT116 | 26.47 |
|  | HT29 | 99.03 |
|  | SW620 | 8.70 |
|  | HCT15 | 370.00 |

Various cancer cell lines were treated with 10^-5^-10^1^ μM YM155 for 48 hours.
